# Supplementary material for: Dishevelled-3 conformation dynamics analyzed by FRET-based biosensors reveals a key role of casein kinase 1
Source: Nat Commun. 2019 Apr 18;10:1804. doi: 10.1038/s41467-019-09651-7 (PMC6472409; doi:10.1038/s41467-019-09651-7)
Supplement: Supplementary file 1 — Reporting Summary [file 41467_2019_9651_MOESM1_ESM.pdf]

nature research

Corresponding author(s): DBPR NCOMMS-18-05111A-Z
Vitezslav Bryja

Last updated by author(s): Mar 11, 2019

Reporting Summary

Nature Research wishes to improve the reproducibility of the work that we publish. This form provides structure for consistency and transparency in reporting. For further information on Nature Research policies, see Authors & Referees and the Editorial Policy Checklist.

Statistics

For all statistical analyses, confirm that the following items are present in the figure legend, table legend, main text, or Methods section.

n/a	Confirmed	
☐	☒	The exact sample size (*n*) for each experimental group/condition, given as a discrete number and unit of measurement
☐	☒	A statement on whether measurements were taken from distinct samples or whether the same sample was measured repeatedly
☐	☒	The statistical test(s) used AND whether they are one- or two-sided<br>*Only common tests should be described solely by name; describe more complex techniques in the Methods section.*
☐	☒	A description of all covariates tested
☐	☒	A description of any assumptions or corrections, such as tests of normality and adjustment for multiple comparisons
☐	☒	A full description of the statistical parameters including central tendency (e.g. means) or other basic estimates (e.g. regression coefficient) AND variation (e.g. standard deviation) or associated estimates of uncertainty (e.g. confidence intervals)
☐	☒	For null hypothesis testing, the test statistic (e.g. *F*, *t*, *r*) with confidence intervals, effect sizes, degrees of freedom and *P* value noted<br>*Give P values as exact values whenever suitable.*
☒	☐	For Bayesian analysis, information on the choice of priors and Markov chain Monte Carlo settings
☒	☐	For hierarchical and complex designs, identification of the appropriate level for tests and full reporting of outcomes
☒	☐	Estimates of effect sizes (e.g. Cohen's *d*, Pearson's *r*), indicating how they were calculated

Our web collection on statistics for biologists contains articles on many of the points above.

Software and code

Policy information about availability of computer code

Data collection	pCLAMP 8.0, Gromacs 5.1.2 simulation package, Vilber FUSION software (Western blotting), FluoView FV1000, Zeiss ZEN 2.0
Data analysis	Origin 7.0, Gromacs 5.1.2 simulation package, MODELLER, Microsoft Excel 2007, GraphPad 6, BioEdit 7.2.5, ImageJ 1.52

For manuscripts utilizing custom algorithms or software that are central to the research but not yet described in published literature, software must be made available to editors/reviewers. We strongly encourage code deposition in a community repository (e.g. GitHub). See the Nature Research guidelines for submitting code & software for further information.

Data

Policy information about availability of data

All manuscripts must include a data availability statement. This statement should provide the following information, where applicable:

- Accession codes, unique identifiers, or web links for publicly available datasets
- A list of figures that have associated raw data
- A description of any restrictions on data availability

Data Availability
Data supporting the findings of this manuscript are available from the corresponding author upon reasonable request. A reporting summary for this Article is available as a Supplementary Information file.
The source data underlying Figs. 1d, 2a, 2c-d, 3e, 3g-h, 3j, 4a, 4c-d, 5b-e, 6h, 6j, 7e-f, 8b-d and Supplementary Figs 1c, 1e, 2a-b, 4a-d, 4f, 7b are provided as a Source Data file. All uncropped Western blots with protein marker are provided as a Source Data file.
The mass spectrometry proteomics data have been deposited to the ProteomeXchange Consortium via the PRIDE75 partner repository with the dataset identifier PXD013085.
Figures were deposited in figshare depository possessing the identifier DOI 10.6084/m9.figshare.7856168

Field-specific reporting

Please select the one below that is the best fit for your research. If you are not sure, read the appropriate sections before making your selection.

☒ Life sciences ☐ Behavioural & social sciences ☐ Ecological, evolutionary & environmental sciences

For a reference copy of the document with all sections, see nature.com/documents/nr-reporting-summary-flat.pdf

Life sciences study design

All studies must disclose on these points even when the disclosure is negative.

Sample size	No sample size calculation was performed.
Data exclusions	No data were excluded.
Replication	All attempts to replicate the results were succesfull.
Randomization	Randomization is not relevant to our study.
Blinding	Blinding was applied in the immunocytochemistry analyses; identity of the samples was disclosed only after counting.

Reporting for specific materials, systems and methods

We require information from authors about some types of materials, experimental systems and methods used in many studies. Here, indicate whether each material, system or method listed is relevant to your study. If you are not sure if a list item applies to your research, read the appropriate section before selecting a response.

Materials & experimental systems

n/a	Involved in the study	
☐	☒	Antibodies
☐	☒	Eukaryotic cell lines
☒	☐	Palaeontology
☐	☒	Animals and other organisms
☒	☐	Human research participants
☒	☐	Clinical data

Methods

n/a	Involved in the study	
☒	☐	ChIP-seq
☒	☐	Flow cytometry
☒	☐	MRI-based neuroimaging

Antibodies

Antibodies used	anti-FLAG M2 (Sigma-Aldrich, #F1804), anti-FLAG (Sigma-Aldrich, #F7425), anti-DVL3 (Santa Cruz, #sc-8027), anti-DVL3 (Cell signaling, #cs-3218S), anti-DVL2 (Cell signaling, #cs-3216S), anti-CK1ε (BD Biosciences, #610445), anti-CK1ε (Santa Cruz, #sc-6471), anti-GFP (Fitzgerald, #20R-GR-011), anti-LRP6 pS1490 (Cell signaling, #cs-2568), anti-tubulin (Sigma-Aldrich, #T6199), anti-DVL3 phospho S643 (Bernatik et al., 2013), and anti-DVL3 phospho S697 (Cervenka et al., 2016), anti-RFP (Chromotek, #5h9).
Validation	According to the manufacture's instruction or published data.

Eukaryotic cell lines

Policy information about cell lines

Cell line source(s)	HEK293 wt and derived cell lines.
Authentication	HEK293 wt were buyed at ATCC company (ATCC-CRL-11268).
Mycoplasma contamination	We confirm that we tested Mycoplasma contamination of all used cell lines.
Commonly misidentified lines (See ICLAC register)	*Name any commonly misidentified cell lines used in the study and provide a rationale for their use.*

Animals and other organisms

Policy information about studies involving animals; ARRIVE guidelines recommended for reporting animal research

Laboratory animals	Xenopus laevis
Wild animals	None.
Field-collected samples	None.
Ethics oversight	The work with Xenopus laevis was carried out according to the German animal use and care law (Das Tierschutzgesetz) and approved by the local authorities and committees (animal care and housing approval: I/39/EE006, Veterinäramt Erlangen; animal experiments approval: 54-2532.2-8/08, German state administration Bavaria/Regierung von Mittelfranken).

Note that full information on the approval of the study protocol must also be provided in the manuscript.
