# Supplementary material for: Dishevelled-3 conformation dynamics analyzed by FRET-based biosensors reveals a key role of casein kinase 1
Source: Nat Commun. 2019 Apr 18;10:1804. doi: 10.1038/s41467-019-09651-7 (PMC6472409; doi:10.1038/s41467-019-09651-7)
Supplement: Supplementary file 2 — Supplementary Information [file 41467_2019_9651_MOESM2_ESM.pdf]

Supplementary information to:

Dishevelled-3 protein conformation dynamics analyzed by FRET-based biosensors reveals a key role of casein kinase 1.

by Harnoš et al.

- Contains 7 Supplementary Figures and 1 Supplementary Table

Supplementary Figure 1.

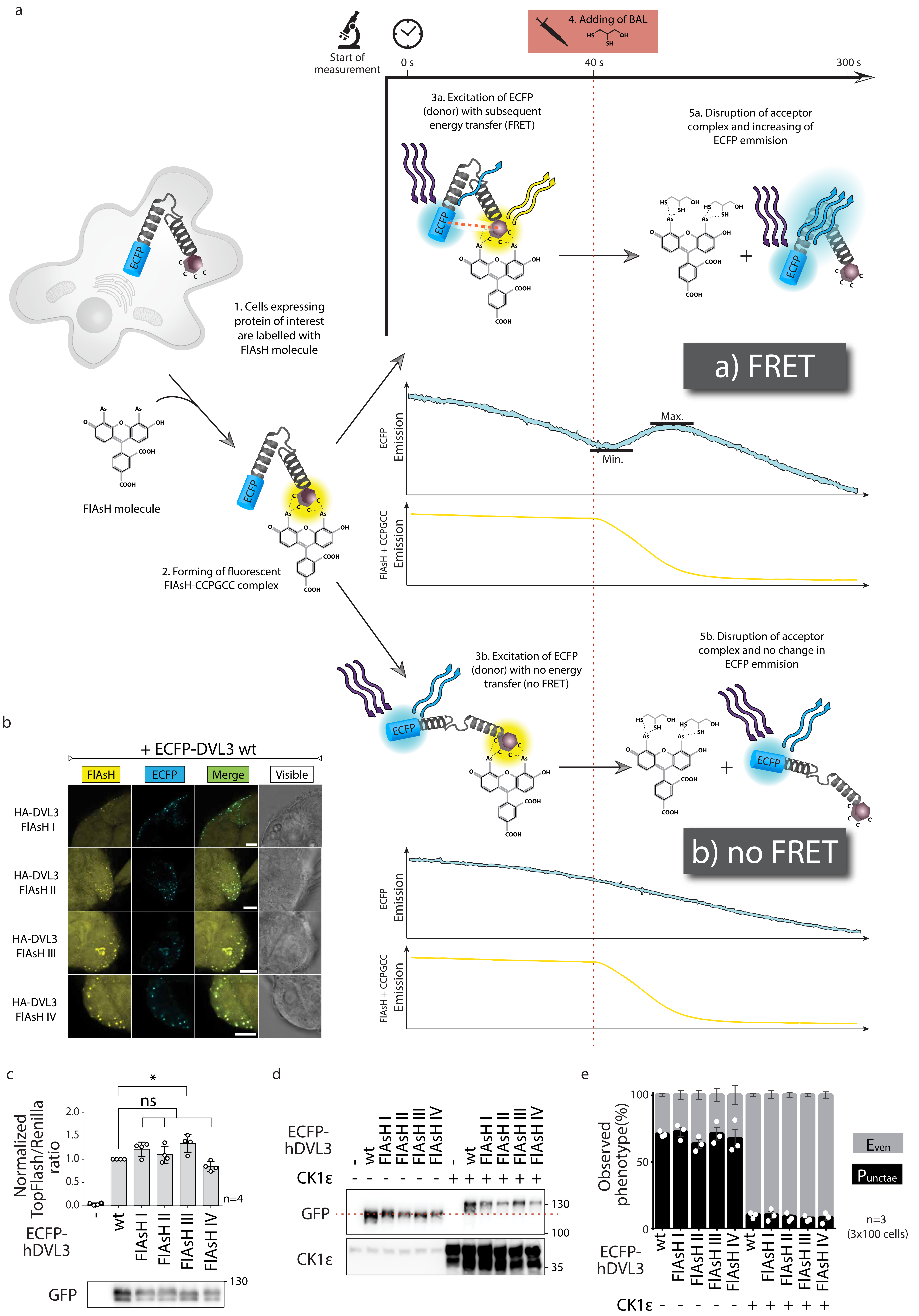

Supplementary Figure 1 DVL3 FlAsH sensors are functionally identical to DVL3 wt

a) Detailed workflow of FlAsH-based FRET in vivo approach. **b)** Representative example of cells used for analysis of the intermolecular FRET efficiency (scale bars, 10 µm). **c-e)** Investigation of biological properties of wild-type ECFP-DVL3 and four ECFP-DVL3 FlAsH sensors by analysis of c) activity of Wnt/β-catenin downstream signaling monitored by Dual Luciferase TopFlash/Renilla Reporter Assay, d) CK1ε-dependent DVL electrophoretic mobility shift assay, and e) their subcellular localization (scale bars, 10 µm), all performed in HEK DVL1-2-3-/- cell line. Data in c) and e) are depicted as Mean ± S.D. {punctae phenotype in e) plotted as white dots}, number of repeats is shown individually. Data in c) were analyzed by One-way ANOVA test with Gaussian distribution; Tukey post test was used for statistical analysis (*, $p \leq 0.05$; **, $p \leq 0.01$; ***, $p \leq 0.001$, ****, $p \leq 0.0001$; ns, not significant, $p > 0.05$).

Supplementary Figure 2.

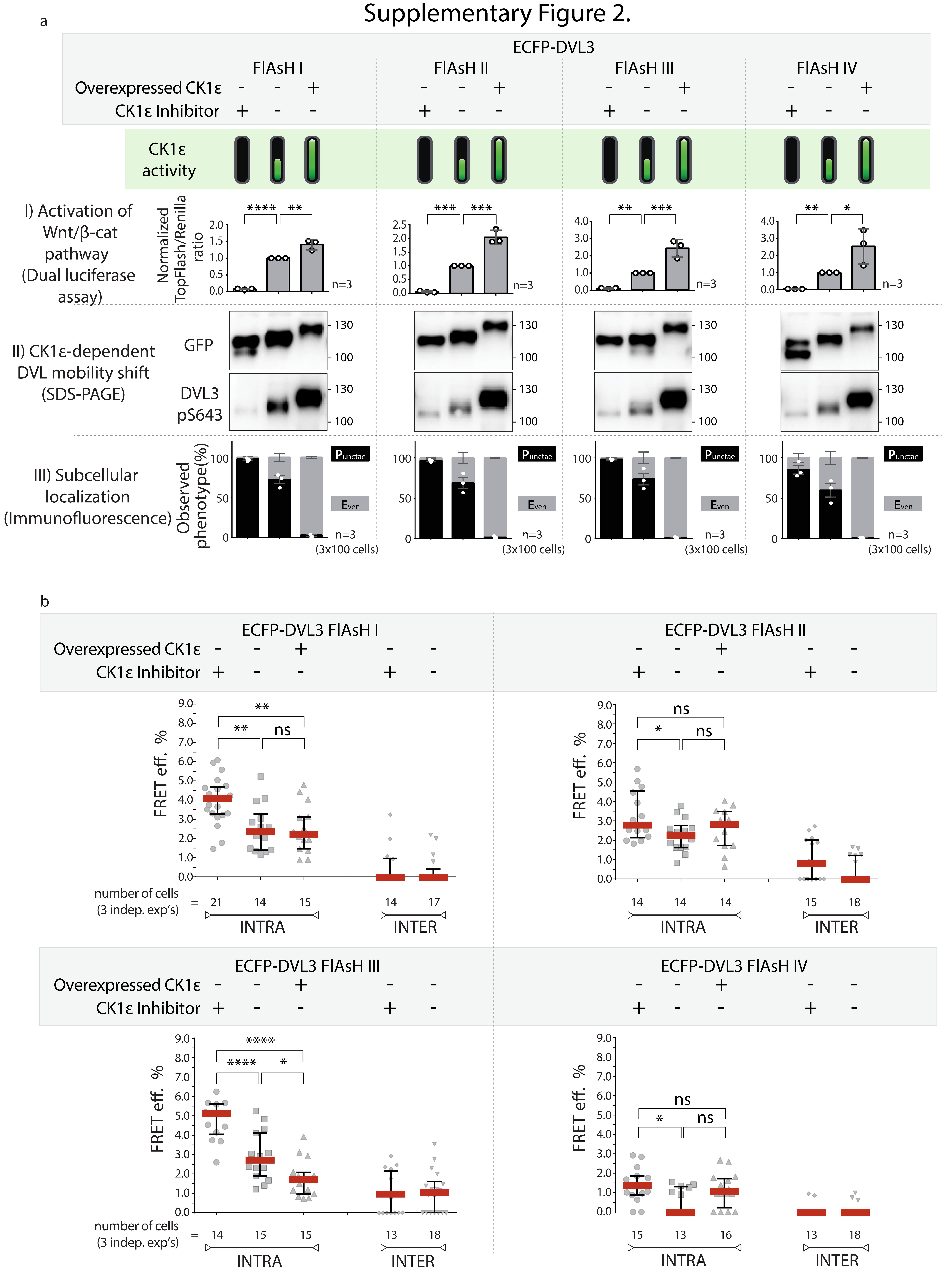

Supplementary Figure 2 Effects of the CK1 activity/levels on DVL3 FlAsH sensors

a) Detailed analysis of CK1ε activity or levels on ECFP-DVL3 FlAsH I-IV sensors monitored by Dual luciferase assay, SDS-PAGE and immunofluorescence in HEK293 wild-type cells. **b)** Detailed measurement of inter- and intramolecular FRET efficiency of DVL3 FlAsH sensors I-IV in HEK293 wild-type cells. Data in a) from Dual luciferase assay and immunofluorescence (punctae phenotype plotted as white dots) are depicted as Mean ± S.D., data in b) as Median ± interquartile range and altogether were analyzed by One-way ANOVA test with Gaussian distribution; Tukey post test was used for statistical analysis (*, $p \leq 0.05$; **, $p \leq 0.01$; ***, $p \leq 0.001$, ****, $p \leq 0.0001$; ns, not significant, $p > 0.05$).

Supplementary Figure 3.

human DVL3

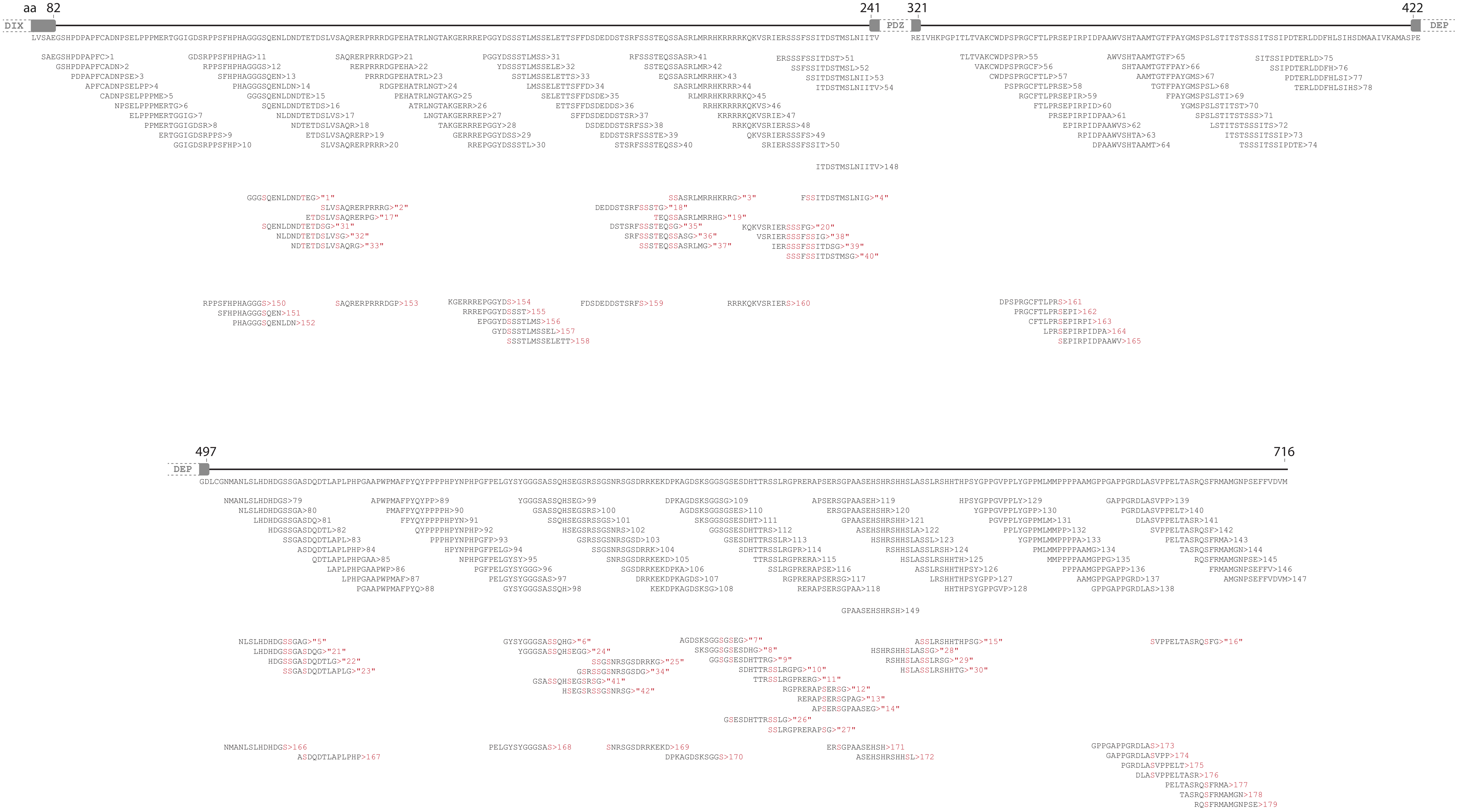

Supplementary Figure 3 Design of the peptide array

Peptide sequences from the DVL3 unstructured parts used in the peptide array and their mapping to human DVL3 is indicated. Non-modified peptides are numbered in grey, phosphorylated peptides in red where phosphorylated serine residues are denoted in red. Numbering of the peptides is arbitrary.

Supplementary Figure 4.

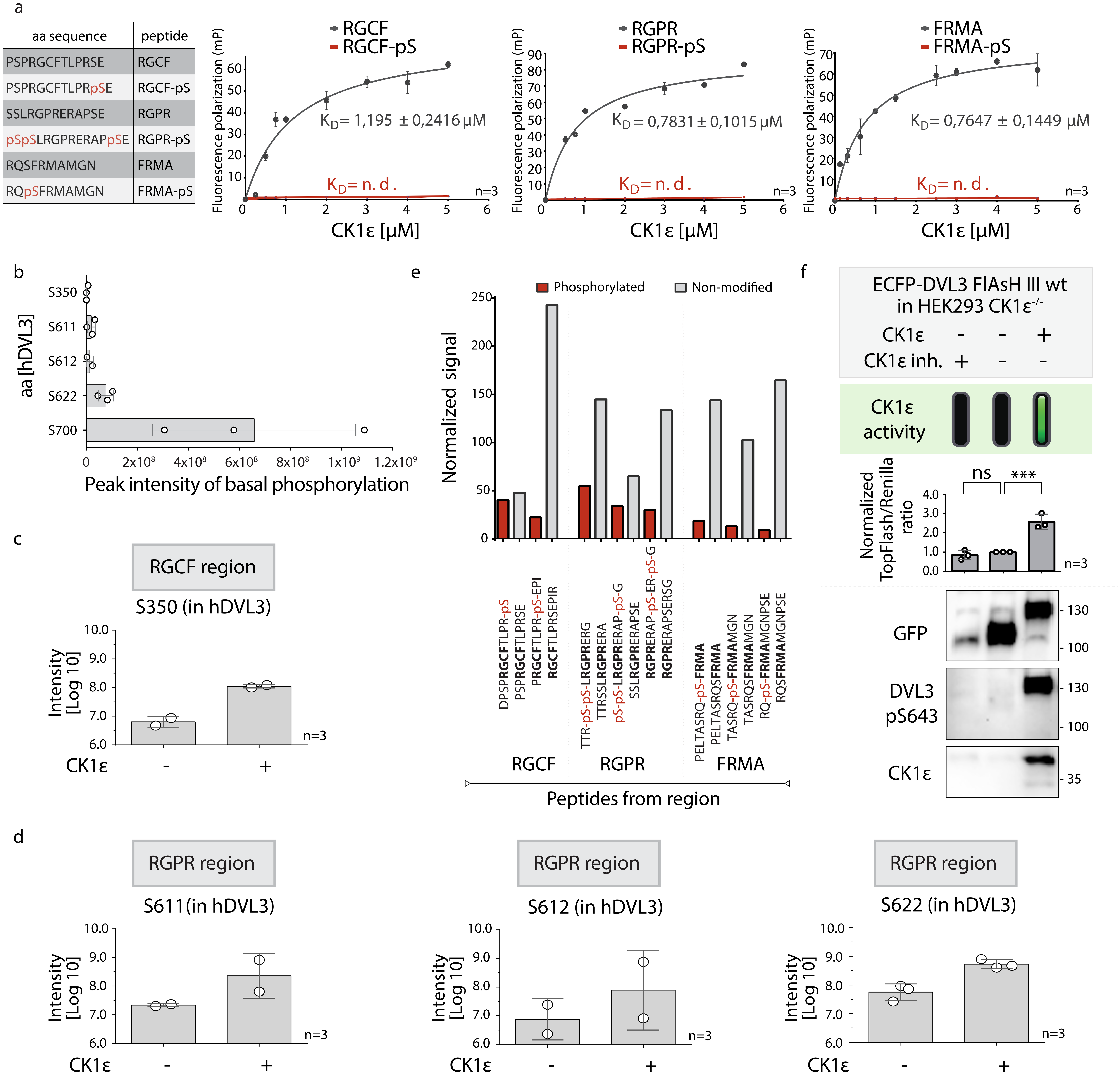

Supplementary Figure 4 Further investigation of DVL3 regions interacting with CK1ε

a) The design and synthesis of the DVL3-derived non-modified and phosphorylated FITC-variants of synthetic peptides, which were used for fluorescent anisotropy (FA) assay. **b)** MS/MS-based analysis of basal phosphorylation showed as intensity of peak corresponding to peptides with the phosphorylated serines in RGCF (S350), RGPR (S611, S612 and S622) and FRMA (S700) regions of DVL3. **c, d)** MS/MS-based analysis of phosphorylation of serine residues present in c) RGCF, and d) RGPR regions of DVL3. FLAG-DVL3 was overexpressed with/without CK1ε in HEK293 cells, immunoprecipitated and phosphorylation in the indicated sites was analyzed by MS/MS approach. Values in c, d show absolute intensity of phosphorylated peptides plotted on a log10 scale. The detection limit is approximately 1.10^6, i.e. 6.0. Individual datapoints represent biological replicates. **e)** Comparison of signals from non-modified (i.e. non-phosphorylated) and phosphorylated peptides derived from peptide array screening (peptides from RGCF, RGPR, FRMA regions are shown). pS means phosphoryleated serine residue and central four amino acids sequences are highilighted in orange. **f)** Detailed

analysis of CK1ε activity or levels on ECFP-DVL3 FlAsH III sensor monitored by Dual luciferase assay and by SDS-PAGE in HEK293 CK1ε-/- cells. Data in f) Dual luciferase assay represent mean ± S.D. and were analyzed by One-way ANOVA test with Gaussian distribution; Tukey post test was used for statistical analysis (*, $p \leq 0.05$; **, $p \leq 0.01$; ***, $p \leq 0.001$, ****, $p \leq 0.0001$; ns, not significant, $p > 0.05$).

Supplementary Figure 5.

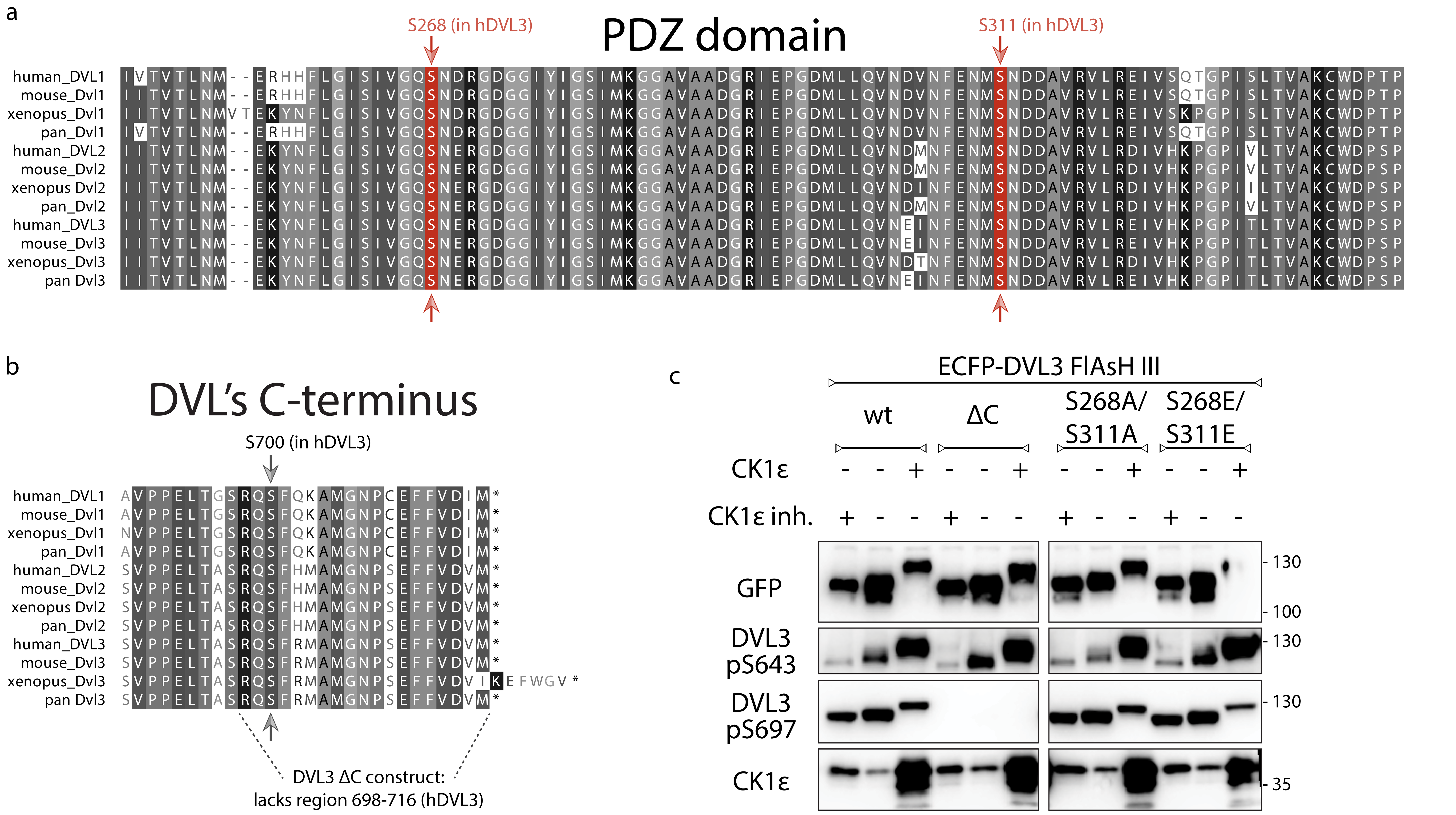

Supplementary Figure 5 Additional analyses of the C-terminus-PDZ domain interaction

a) Multiple sequence alignment of selected Dvl/DVL sequences from different organisms showing similarity of PDZ domain is shown. Two serine residues corresponding to S268 and S311 in DVL3 are highlighted by red arrow. Residues with >80% similarity are highlighted. **b)** Multiple sequence alignment of selected Dvl/DVL C-terminus sequences from different organisms is shown. Serine residue corresponding to S700 in DVL3 is highlighted by grey arrow. Residues with >80% similarity are highlighted. **c)** Investigation of CK1ε activity or levels with the CK1 inhibitor or CK1ε overexpression on PDZ- and C-terminal- mutants of ECFP-DVL3 FlAsH III sensor by SDS-PAGE in HEK293 wild-type cells.

Supplementary Figure 6.

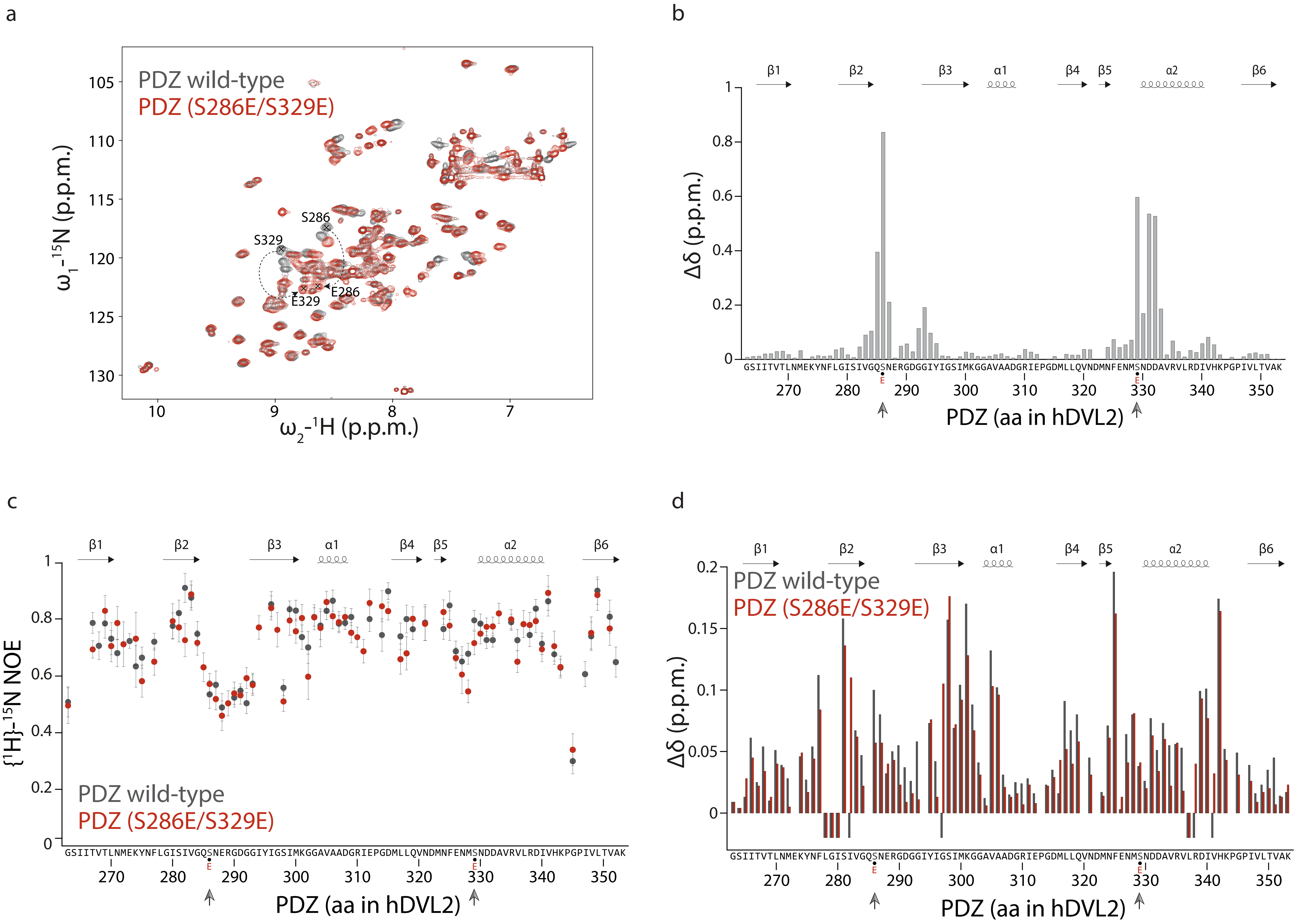

Supplementary Figure 6 Role of S268/S311 in the C-terminus-PDZ domain interaction

a) 1H, 15N HSQC spectra of PDZ wild-type (grey) and phosphorylation-mimicking mutant S286E+S329E (red). All amide frequencies have been assigned unambiguously using 4D spectra. The serine cross-peaks of wild-type and corresponding glutamic acids cross-peaks of the mutant are marked. **b)** Weighted chemical shift differences between PDZ wild-type and S286E+S329E mutant versus the protein sequence. **c)** {1H}-15N heteronuclear NOE values of PDZ wild-type (grey) and S286E+S329E mutant (red) versus the protein sequence. The error for each point was estimated from the noise level. **d)** Weighted chemical shift perturbations between free and DVL C-terminal peptide bound state for PDZ wild-type (grey) and S286E+S329E mutant (red) versus the protein sequence. In plots b-d, the PDZ secondary structure is indicated at the top and the mutation sites are marked below with arrows.

Supplementary Figure 7.

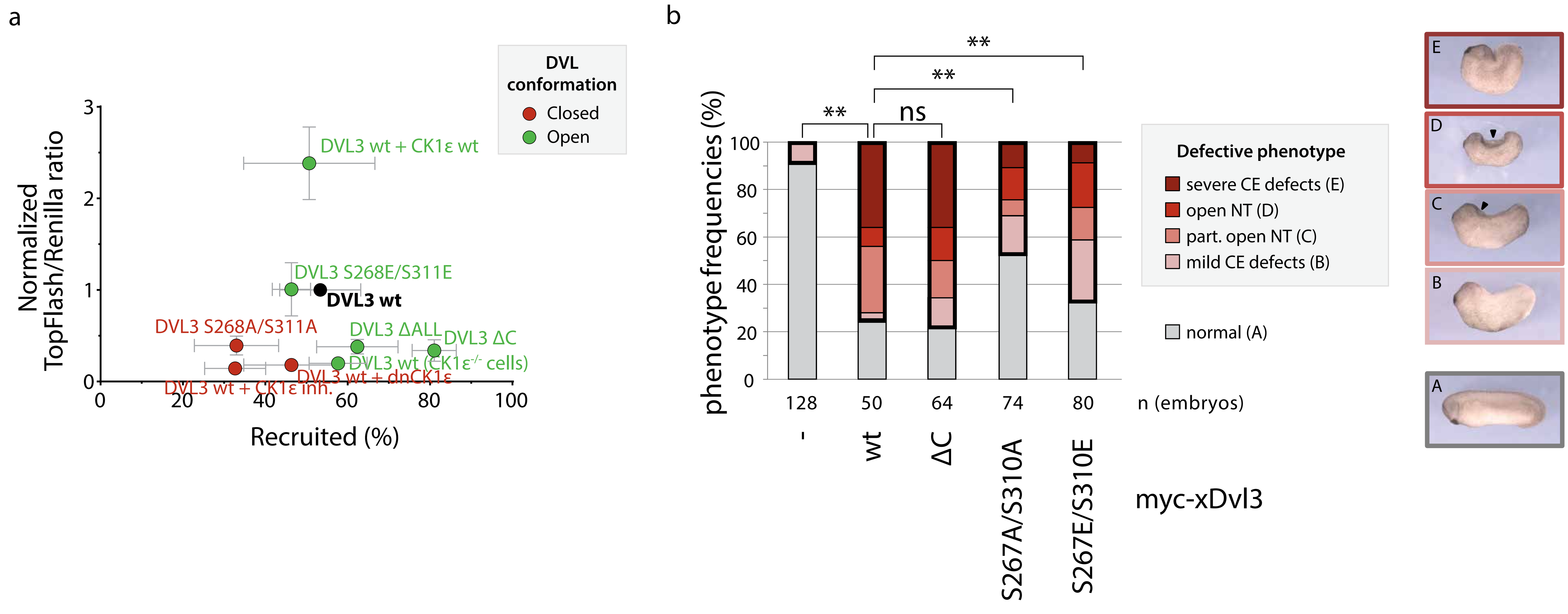

Supplementary Figure 7 Additional investigation of DVL conformations

a) Data from DVL membrane recruitment and the TopFlash assay obtained in Fig. 8c-d were plotted in the 2D graph. Red color indicates the closed conformation, green indicates open. Error bars show S.D. for each parameter. % of recruited is a sum of partially recruited and fully recruited in Fig. 8c. **b)** In vivo analysis of convergent extension defects in the *Xenopus* embryos. Overexpression of xDvl3 causes Convergent Extension (CE) defects due to the activation of non-canonical Wnt/planar cell polarity (PCP) pathway. CE defects can be classified into four categories and the representative example of affected embryo in each category is shown (on the right). The capacity of selected variants of myc-xDvl3 to induce CE defects in *Xenopus* was analyzed. Data in b) were analyzed by One-way ANOVA test with Gaussian distribution; Tukey post test was used for statistical analysis (*, $p \leq 0.05$; **, $p \leq 0.01$; ***, $p \leq 0.001$, ****, $p \leq 0.0001$; ns, not significant, $p > 0.05$).

Supplementary Table 1.

List of the primers used for the site-directed mutagenesis.

Xenopus

pCS2 myc-Dvl3 ΔALL

>Delta 1 xDsh3 forw
actgttgccaagtgctgggatccaataagaccgattgatcca

>Delta 1 xDsh3 rev
tggatcaatcggtcttattggatcccagcacttggcaacagt

>Delta 2 xDsh3 forw
gagtccgatcataccacgcgtcgttcagttccagcaagtgaa

>Delta 2 xDsh3 rev
TTCACTTGCTGGAACTGAACGACGCGTGGTATGATCGGACTC

>Delta 3 xDsh3 forw
ccacctgaactcaccgctagtaattctggggtgtttgacttt

>Delta 3 xDsh3 rev
AAAGTCAAACACCCCAGAATTACTAGCGGTGAGTTCAGGTGG

pCS2 myc-Dvl3 S267A/S310A

>S268A xDsh3 forw
atcagcattgtaggacaaGCcaatgaacgtggggatgga

>S268A xDsh3 rev
TCCATCCCCACGTTCATTGGCTTGTCCTACAATGCTGAT

>S311A xDsh3 forw
accaattttgagaacatggccaatgatgatgcagtgcgg

>S311A xDsh3 rev
ccgcactgcatcatcattggccatgttctcaaaattggt

pCS2 myc-Dvl3 S267E/S310E

>S268E xDsh3 forw
atcagcattgtaggacaaGAGaatgaacgtggggatgga

>S268E xDsh3 rev
TCCATCCCCACGTTCATTCTCTTGTCCTACAATGCTGAT

>S311E xDsh3 forw
accaattttgagaacatggagaatgatgatgcagtgcgg

>S311E xDsh3 rev
ccgcactgcatcatcattctccatgttctcaaaattggt

pCS2 myc-Dvl3 ΔC (aa 1-692)

>dFRMA-C7 xDsh3 FOR
gtaccacctgaactcaccgctagttagaACTATAGTGAGTCGTATTAC

Human

pCDNA3.1 FLAG-hDVL3 ΔALL

>Delta 1 hDVL3 forw
CTGACTGTAGCCAAGTGCTGGGACCCCATCCGGCCCATTGACCCTGCG

>Delta 1 hDVL3 rev
CGCAGGGTCAATGGGCCGGATGGGGTCCCAGCACTTGGCTACAGTCAG

>Delta 2 hDVL3 forw
AGCGAATCGGACCACACCACACGCCGCTCAGGGCCGGCGGCAAGCGAG

>Delta 2 hDVL3 rev
CTCGCTTGCCGCCGGCCCTGAGCGGCGTGTGGTGTGGTCCGATTCGCT

>Delta 3 hDVL3 forw
GTGCCCCCGGAACTGACCGCCAGCCCCAGTGAGTTCTTTGTGGATGTG

>Delta 3 hDVL3 rev
CACATCCACAAAGAACTCACTGGGGCTGGCGGTCAGTTCCGGGGGCAC

pCDNA3.1 FLAG-hDVL3 S268A/S311A

>Dvl3 S268A forw
ATCTCCATTGTGGACCAAGCCAACGAGCGTGGTGACGGC

>Dvl3 S268A rev
GCCGTCACCACGCTCGTTGGCTTGGTCCACAATGGAGAT

>DVL3 S311A for
ATCAACTTTGAGAACATGGCTAATGACGATGCAGTCCGG

>DVL3 S311A rev
CCGGACTGCATCGTCATTAGCCATGTTCTCAAAGTTGAT

pCDNA3.1 FLAG-hDVL3 S268E/S311E

>Dvl3 S268E forw
ATCTCCATTGTGGACCAAGAGAACGAGCGTGGTGACGGC

>Dvl3 S268E rev
GCCGTCACCACGCTCGTTCTCTTGGTCCACAATGGAGAT

>Dvl3 S311E forw
ATCAACTTTGAGAACATGGAGAATGACGATGCAGTCCGG

>Dvl3 S311E rev
CCGGACTGCATCGTCATTCTCCATGTTCTCAAAGTTGAT

pCDNA3.1 FLAG-hDVL3 ΔC (aa 1-697)

>Delta C7 from delta 3 forw
CCCCCGGAACTGACCGCCAGCTGAGAATTCGCCCTTTTCGGA

Human

pdECFP hDvl3 FlAsH III ΔALL

>Delta 1 hDVL3 forw
CTGACTGTAGCCAAGTGCTGGGACCCCATCCGGCCCATTGACCCTGCG

>Delta 1 hDVL3 rev
CGCAGGGTCAATGGGCCGGATGGGGTCCCAGCACTTGGCTACAGTCAG

>Delta 2 hDVL3 forw
AGCGAATCGGACCACACCACACGCCGCTCAGGGCCGGCGGCAAGCGAG

>Delta 2 hDVL3 rev
CTCGCTTGCCGCCGGCCCTGAGCGGCGTGTGGTGTGGTCCGATTCGCT

>Delta 3 hDVL3 forw
GTGCCCCCGGAACTGACCGCCAGCCCCAGTGAGTTCTTTGTGGATGTG

>Delta 3 hDVL3 rev
CACATCCACAAAGAACTCACTGGGGCTGGCGGTCAGTTCCGGGGGCAC

pdECFP hDvl3 FlAsH III S268A/S311A

>Dvl3 S268A forw
ATCTCCATTGTGGACCAAGCCAACGAGCGTGGTGACGGC

>Dvl3 S268A rev
GCCGTCACCACGCTCGTTGGCTTGGTCCACAATGGAGAT

>DVL3 S311A for
ATCAACTTTGAGAACATGGCTAATGACGATGCAGTCCGG

>DVL3 S311A rev
CCGGACTGCATCGTCATTAGCCATGTTCTCAAAGTTGAT

pdECFP hDvl3 FlAsH III S268E/S311E

>Dvl3 S268E forw
ATCTCCATTGTGGACCAAGAGAACGAGCGTGGTGACGGC

>Dvl3 S268E rev
GCCGTCACCACGCTCGTTCTCTTGGTCCACAATGGAGAT

>Dvl3 S311E forw
ATCAACTTTGAGAACATGGAGAATGACGATGCAGTCCGG

>Dvl3 S311E rev
CCGGACTGCATCGTCATTCTCCATGTTCTCAAAGTTGAT

pdECFP hDvl3 FlAsH III ΔC (aa 1-697)

>Delta C7 from delta 3 forw
CCCCCGGAACTGACCGCCAGCTGAGAATTCGCCCTTTTCGGA

Human

pDONR221 hDvl3 FlAsH I

>Flash I. expanded for
tggagaccaccagcttctttTGTTGCCCGGGCTGCTGTgactcagatgaggatgactc

>Flash I. expanded rev
gagtcatcctcatctgagtcACAGCAGCCCGGGCAACAaaagaagctggtggtctcca

pDONR221 hDvl3 FlAsH II

Flash II. expanded for
tcacctccaccagctcctccTGTTGCCCGGGCTGCTGTatcaccagttccatccctga

Flash II. expanded rev
tcagggatggaactggtgatACAGCAGCCCGGGCAACAggaggagctggtggaggtga

pDONR221 hDvl3 FlAsH III

Flash III. expanded for
gcgccagcagtcagcacagcTGTTGCCCGGGCTGCTGTgaaggcagtcggagcagtgg

Flash III. expanded rev
cactgctccgactgccttcACAGCAGCCCGGGCAACAgctgtgctgactgctggcgc

pDONR221 hDvl3 FlAsH IV

Flash IV. supernew for
gagttctttgtggatgtgatgTGTTGCCCGGGCTGCTGTtaggACCCAGCTTTCTTGTAC

Flash IV. supernew
rev
GTACAAGAAAGCTGGGTcctaACAGCAGCCCGGGCAACAcatcacatccacaaagaactc

Human

pETM-11 MAGS-HEPR wt pLipK-PDZ wt (hDvl2 PDZ aa 265-361) S286E/S313E

>S268E Dvl2 forw
ATCTCCATTGTTGGCCAGGAGAATGAGCGGGGAGACGGA

>S268E Dvl2 rev
TCCGTCTCCCCGCTCATTCTCCTGGCCAACAATGGAGAT

>Dvl2 S311E forw
ATCAACTTTGAGAACATGGAGAATGACGATGCAGTCCGG

>Dvl2 S311E rev
CCGGACTGCATCGTCATTCTCCATGTTCTCAAAGTTGAT
